# Influence of the pesticide flupyradifurone on mobility and physical condition of larval green lacewings

**DOI:** 10.1038/s41598-023-46135-7

**Published:** 2023-11-13

**Authors:** Leonie Scheibli, Tabita Elsenhans, Harald Wolf, Torben Stemme, Sarah Elisabeth Pfeffer

**Affiliations:** Institute of Neurobiology, Ulm University, Albert-Einstein-Allee 11, 89081 Ulm, Germany

**Keywords:** Neurophysiology, Agroecology, Behavioural ecology, Entomology

## Abstract

Global pesticide use in agriculture is one reason for the rapid insect decline in recent years. The relatively new pesticide flupyradifurone is neurotoxic to pest insects but considered harmless to bees according to previous risk assessments. With this study, we aim to investigate lethal and sublethal effects of flupyradifurone on larvae of the beneficial arthropod *Chrysoperla carnea*. We treated the animals orally with field-realistic concentrations of flupyradifurone and examined lethality as well as effects on condition, mobility and locomotion. For the lethal dose 50, we determined a value of > *120–200 *ng/mg (corresponding to a mean amount of 219 ng/larva) after 168 h. Abnormal behaviors such as trembling and comatose larvae were observed even at the lowest concentration applied (> *0–20 *ng/mg, 59 ng/larva). Mobility analysis showed impaired activity patterns, resulting in acute hypoactivity at all pesticide concentrations and time-delayed hyperactivity in larvae treated with > *40–60 *ng/mg (100 ng/larva) and > *80–100* ng/mg (120 ng/larva), respectively. Even locomotion as a fundamental behavioral task was negatively influenced throughout larval development. In conclusion, our results demonstrate that flupyradifurone impacts life and survival of lacewing larvae and may pose—despite its status as bee-friendly—a major threat to insect fauna and environment.

## Introduction

Insects provide a variety of ecosystem services indispensable to mankind, such as soil and freshwater functions, pollination, biological pest control or as food web components^1,2^. However, a massive decline in insects has been documented in recent decades, which poses a major threat to future generations^2–4^. A long-term study covering 27 years revealed a dramatic loss of 76% biomass in flying insects^5^. These data have been gathered in protected areas of Germany and, thus, may even underrate the actual loss of flying insects in agricultural regions. Sánchez-Bayo and Wyckhuys^6^ concluded that one third of all insect species worldwide are threatened by extinction. Agricultural intensification and associated use of pesticides are one of the main causes of biodiversity loss^6–8^.

It has indeed been demonstrated that pesticide use has detrimental effects on insects^9^. In particular neonicotinoids, agonists of nicotinic acetylcholine receptors (nAChR), negatively affect insect survival^10^. Beside their lethal impacts, neonicotinoids could have sublethal effects like impaired learning and memory, abnormal behavior and impact on mobility^11–16^. Therefore, three neonicotinoids (imidacloprid, thiamethoxam and clothianidin) have been banned by the European Union^17–19^. These restrictions, together with insect pest resistance, drive the development of new substances^20^. A rather new pesticide, the butenolide flupyradifurone, has a similar mode of action as neonicotinoids, reversibly binding to insect nAChRs. Flupyradifurone is considered as potentially “bee-safe” by risk assessments, although it has effects similar to those of neonicotinoids^21–25^.

Current risk assessments focus on lethal toxicity, although there is evidence that sublethal effects of pesticides are underestimated regarding insect survival^9,26^. It is not sufficient to examine lethal effects only, especially as lethal dose 50 (LD_50_)-values are highly variable in flupyradifurone as well as in neonicotinoids. Even in a single species, *Apis mellifera*, LD_50_-values range from 1200 ng/bee up to 6823 ng/bee for flupyradifurone^21,27^ from 3.7 to 90.1 ng/bee for imidacloprid^28^, and from 3.8 ng/bee up to 25.4 ng/bee for clothianidin^28^. In previous studies mainly honeybees have been investigated, leaving a large data gap concerning other species^12,14,24^. Another limitation of current risk assessments is the use of species-dependent behavioral endpoints to assess pesticide impacts, for example, foraging behavior or proboscis extension reflex^29^. These parameters are not eligible for most other insect species^30^. To obtain a realistic picture of the detrimental effects of pesticides and to take appropriate protective actions, it is decisive to investigate lethal and sublethal effects and to address a broader range of species.

It is crucial to examine pesticide concentrations within field-realistic lethal and sublethal ranges. Species like *Chrysoperla carnea*, often employed in integrated pest management (IPM) strategies^31,32^, face potential risks from pesticides, including flupyradifurone. The diverse plant practices in crops and horticulture, coupled with various flupyradifurone application methods (foliar, drench, drip, seed treatment^33^, contribute to potential contamination of lacewing larvae and adults. Despite limited data on exposure rates for non-hymenopterans like lacewings, we consider our applied flupyradifurone ranges as a field-realistic scenario (for more details see “Discussion” section, chapter “field relevance”).

In this study, we investigated the effects of orally applied flupyradifurone (50–300 ng/individual, corresponding to > 0–200 ng/mg) on larvae of the common green lacewing *C. carnea* (Stephens, 1836). These larvae are beneficial arthropods as they feed on soft-bodied pests and can consequently be used as a biological control agent^34,35^. The aim of our study not only was the examination of lethality but also the analysis of possible sublethal effects of flupyradifurone. Therefore, we examined parameters like physical condition, developmental state, activity state, mobility patterns and locomotion capacity of the animals. Our goal was to use fundamental parameters like the condition of the animals or their mobility to provide comparable data.

## Results

**Figure 1:**
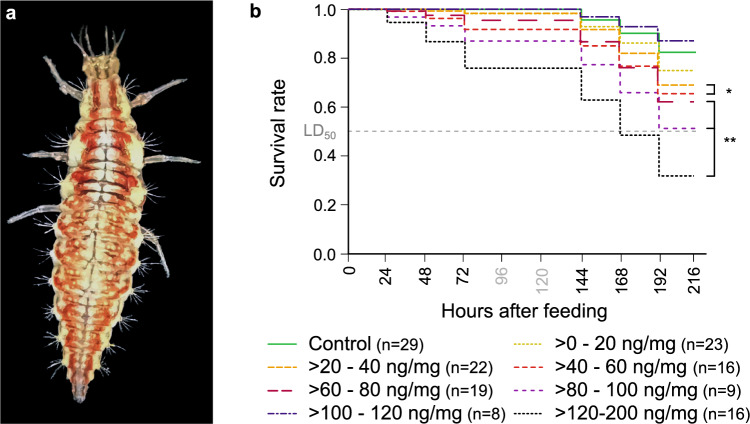
Experimental animal and survival rates after being fed flupyradifurone or control solution. (**a**) Habitus of *C. carnea* larva. (**b**) Lethality was determined at different time points before and after consumption of pesticide or control solution. Observations took place directly before application (0 h) and up to 216 h (9 days) after intoxication. As 96 and 120 h after feeding no observation took place, these time points are depicted in gray. Different colors indicate particular concentration ranges. The dashed gray line represents LD_50_, defined as the dose which kills 50% of the test animals after a certain time. Sample size varies between different treatments (see “Methods” for further information). Statistically significant differences are highlighted with an asterisk (Kaplan–Meier and Log Rank Test, N = 127, *p < 0.05, **p < 0.01). Significant differences in long-term survival were observed in all pesticide-treated groups except the lowest concentration of > *0–20 *ng/mg (and > *100–120 *ng/mg, see results and “Discussion”).

Larvae of *C. carnea* (Fig. 1a) were fed with four concentrations of flupyradifurone (50, 100, 200, 300 ng) and afterwards categorized in seven groups regarding the amount of pesticide fed per body mass (> *0–20;* > *20–40;* > *40–60;* > *60–80;* > *80–100;* > *100–120 and* > *120–200* ng/mg, compare “Methods”). We studied lethal and sublethal impacts of the pesticide concentrations in comparison to a control group. Note that sample sizes varied in concentration groups throughout different experiments for several reasons (see “Methods” for more information).

### Survival analysis

We observed the animals for up to 216 h (9 days) after treatment to determine the lethality (Fig. 1b). All concentrations administered, except the lowest concentration, showed a statistically significant difference from the control group (Kaplan–Meier and Log Rank Test, N = 127, *p < 0.05, **p < 0.01). Survival rates ranged from 87% (> *100–120 *ng/mg) to 32% (> *120–200 *ng/mg) on the last day of the observation period. In all pesticide-treated groups, except the > *100–120 *ng/mg group, mortality occurred earlier than in the control. In groups treated with at least *60* ng/mg, the first animals died already 24 h after feeding (control 144 h). After 168 h, we determined an LD_50_-value for the > *120–200 *ng/mg group (corresponding to a mean amount of 219 ng/larva). It is noteworthy that the > *100–120 *ng/mg group had the highest survival rate (87% control: 82%) and all animals survived until 144 h.

### Analysis of physical condition and developmental state

**Figure 2:**
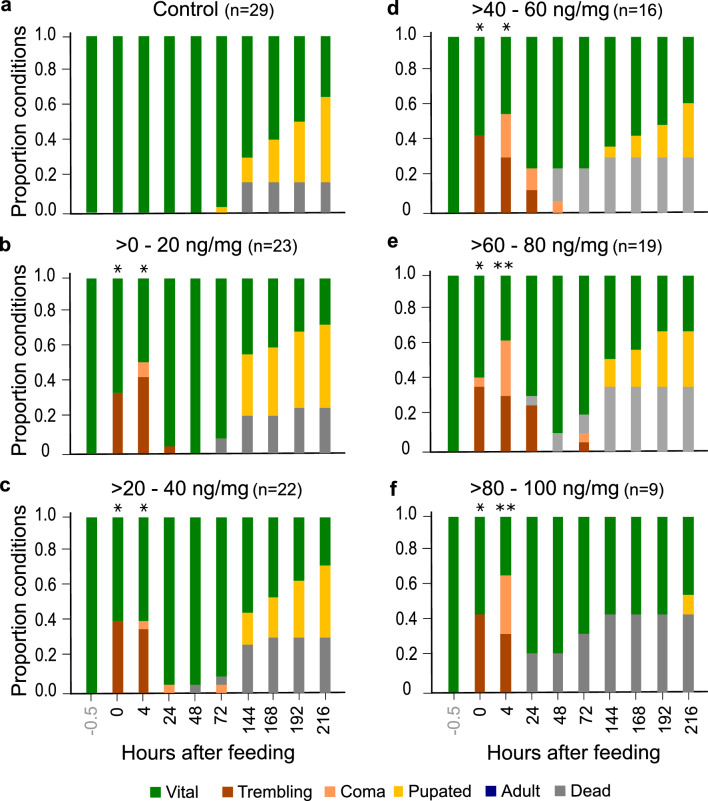
Proportions of conditions and developmental states of larvae after being fed flupyradifurone or control solution. Animals were observed at different time points after feeding (0–216 h) and assigned to one of the following states: *vital, trembling, coma, pupated, adult, dead*. Categories are marked by different colors (bottom legend). Results for different concentration ranges are shown in separate graphs (**a**–**f**). Statistical differences were determined with a Chi-Square-Test. Each concentration was tested against the control group for every observation time, significant differences from controls are featured by asterisks in the pesticide groups (*p < 0.05, **p < 0.01). Sample size states the number of animals observed, even if they were classified as dead in the course of the experiment. Sample size varies between different treatment groups (see “Methods” for further information). Note that the abnormal behaviors “*trembling*” and “*coma*” occurred in all pesticide-treated groups. Additionally, time until pupation increased in animals treated with > *80–100 *ng/mg (compare Supplementary Table S2).

Physical condition and developmental state (Fig. 2) were observed at concentrations of > *0–100 *ng/mg. 50% of the animals treated with a concentration of > *120–200 *ng/mg were dead after 168 h and thus not suitable for long-term observations (see Fig. 1b). We further excluded the groups treated with > *100–120 *ng/mg and > *120–200 *ng/mg in the following experiments since only few animals were able to ingest this amount of pesticide.

Flupyradifurone caused trembling and coma, even at lowest doses, in a concentration-dependent manner (Fig. 2b–f). Directly after pesticide application (0 h), animals were mostly vital or trembling, the latter characterized by uncontrolled lifting of the abdomen or leg shaking (Supplementary Video V1). 4 h after administration, trembling and comatose larvae were observed in all pesticide-treated groups. The lowest number of coma (except for controls) was detected in the > *20–40 *ng/mg group, which contained 4% comatose larvae after 4 h (Fig. 2c), whereas the highest number of 33% was recorded in the > *80–100 *ng/mg group (Fig. 2f). At the lowest concentrations, animals were mostly in a trembling state (44% in > *0–20 *ng/mg group; 36% in > *20–40 *ng/mg group, Fig. 2b, c), if showing any abnormal behavior after 4 h.

The differences compared to controls were statistically significant in all concentration groups 0 h and 4 h after treatment (Chi-Square-Test, 0 h p < 0.05 in all groups, 4 h p < 0.05 for > *0–20 *ng/mg; > *20–40 *ng/mg; > *40–60 *ng/mg and p < 0.01 for > *60–80 *ng/mg and > *80–100 *ng/mg). Abnormal behaviors could be observed up to 72 h after pesticide application. There were no statistically significant differences, however, because the animals had partially recovered to a vital state or died.

First pupated larvae occurred after 72 h in controls (Fig. 2a), whereas pupated animals occurred not until 216 h in the > *80–100 *ng/mg group (Fig. 2f). Flupyradifurone thus tended to retard larval development at high concentrations (Fig. 2f).

### Mobility analysis

#### Trajectories and activity state

**Figure 3:**
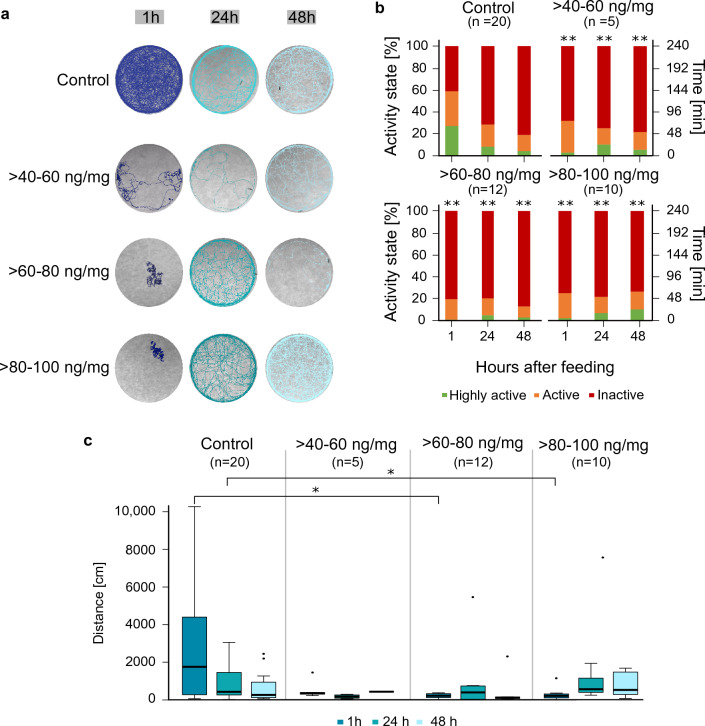
Sample tracks of larvae, activity states and distances traveled during 4 h tracking. (**a**) Trajectories of animals during 4 h recording are shown, particular colors state the different observation periods (1 h, 24 h, 48 h after application). Directly after feeding, all pesticide-treated groups showed hypoactive behavior, which changed to time-delayed hyperactivity in the > *40–60 *ng/mg group as well as in the > *80–100 *ng/mg group at 48 h. (**b**) Activity states of the controls and treatment groups. Depicted are three bars per group representing the observation times 1 h, 24 h and 48 h after consumption. Each bar shows the percentage, and the total time spent in the respective activity state “highly active”, “active” or “inactive” in different colors. The activity state defined the percentage of movement of the body independent of spatial motion of the center point (see “Methods” for further explanation). We conducted a Chi-Square-Test with the absolute data to test for statistical significance. Each concentration was tested against the control group for every observation time, significant results are featured with asterisks in the pesticide groups (**p < 0.01). Sample size states the number of animals observed at the beginning of the observation period (see “Methods” for the varying sample size, see sample size changes within time course due to dead animals in Fig. 4). Control animals got less active with increasing time, while an opposite trend was observed in the treatment group of > *80–100 *ng/mg. (**c**) Distance traveled over the entire 4 h observation period. The four treatment groups are labeled and separated by gray lines, observation periods are marked by different colors. Significant differences are featured with an asterisk (*p < 0.05), Kruskal–Wallis-Test and a Wilcoxon-Test were used for pairwise comparison. Sample size states the number of animals observed at the beginning of the observation period (see “Methods” for the varying sample size, see sample size changes within time course due to dead animals in Fig. 4). Trends of the activity states are backed by the results concerning traveled distances. Here, control animals walked the longest distances during the first observation period and shorter distances after 24 h and 48 h. The highest concentration group exhibited a reversed pattern.

The mobility of the larvae was investigated at three time points after treatment (1 h, 24 h, 48 h) to examine differences regarding traveled paths, activity state and covered distances (Fig. 3). Besides the controls, treatment groups > *40–60 *ng/mg, > *60–80 *ng/mg, > *80–100 *ng/mg were used. These were chosen according to their susceptibility to flupyradifurone shown in the previous experiment.

1 h after application, control animals walked 1,754 ± 586 cm (median ± standard error of the mean (s.e.m.)) on average, during a 4 h period (Fig. 3c). All flupyradifurone-treated animals walked significantly shorter distances, between 349 ± 228 cm (> *40–60 *ng/mg) and 211 ± 101 cm (> *60–80 *ng/mg), showing a hypoactive state directly after feeding compared to controls. Differences between controls and the two highest concentration groups (> *60–80 *ng/mg and > *80–100 *ng/mg) were statistically significant 1 h after insecticide application (Kruskal–Wallis-Test and Wilcoxon-Test *p < 0.05).

The distance covered by the control animals decreased gradually after 24 h and 48 h to 425 ± 192 cm and 257 ± 171 cm, respectively. Activity state analysis showed a similar pattern for the control group, with high activity 27% of the time 1 h after treatment, dropping to a value of 4% after 48 h (Fig. 3b). Note that the activity decrease in the control group is due to normal development and deceleration before pupation^36,37^. Contrary to expectations, the treated larvae covered relatively constant distances over time. Intriguingly, animals treated with > *40–60 *ng/mg and > *80–100 *ng/mg walked more than the control 48 h after feeding (control: 257 ± 171 cm, > *40–60 *ng/mg: 428 ± 2 cm, > *80–100 *ng/mg: 526 ± 1008 cm), exhibiting a hyperactive behavior compared to control animals (Fig. 3c). This trend was also detected in the activity states of the > *80–100 *ng/mg group (48 h, Fig. 3b), where the animals were in a highly active state 10% of the recording time.

#### Mobility patterns

**Figure 4:**
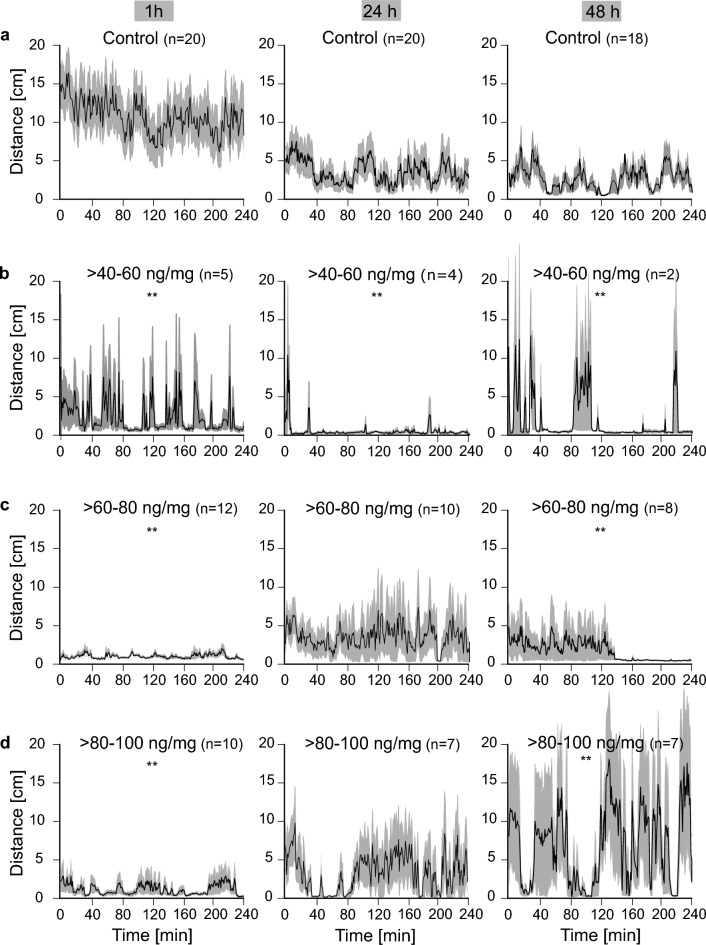
Tracking analysis of *C. carnea* larvae after feeding with flupyradifurone or control solution. The mean covered distance per minute per individual is shown for the controls and the three concentration groups (**a**–**d**), 1 h, 24 h and 48 h after pesticide treatment. The black curve represents the mean covered distance per minute per individual and the gray shadows depict the standard errors of the mean. Sample size varies between different treatment groups (see “Methods” for further information). Significant differences from the control are featured with asterisks in the pesticide groups (**p < 0.01), Kruskal–Wallis-Test and a Wilcoxon-Test were used for the pairwise comparison. Control larvae covered smaller distances with progressing time, but they exhibited clearly higher activity than the pesticide-treated larvae 1 h after feeding. In comparison, all pesticide-treated animals showed hypoactive behavior during first observation period (1 h) with a high amount of low activity periods. As time passed, pesticide-treated larvae became more active and 48 h after feeding, animals treated with >  *40–60* ng/mg or > *80–100* ng/mg of pesticide showed hyperactivity compared to controls.

In a different approach, we focused on the temporal context and recorded the distance covered per minute by larvae during 4 h (Fig. 4). Control animals (Fig. 4a) were highly active 1 h after they had been treated with sugar solution, and walked 6–16 cm/min on average. 24 h after application, the overall activity decreased and the controls walked 1–7 cm/min. 48 h after consumption, the larvae covered only mean distances of 0.5–5 cm/min. This time course reflects the expected larval behavior during development^36,37^.

Pesticide groups covered distinctly shorter distances and walked merely 0–9 cm/min 1 h after administration (Fig. 4b–d, 1 h). It is conspicuous that only animals treated with > *60–80 *ng/mg or > *80–100 *ng/mg flupyradifurone showed a high amount of low activity phases, covering less than 5 cm/min, during first observation period. The same groups showed similar activities as the controls 24 h after feeding, whereas animals fed with the lowest concentration of flupyradifurone showed the least activity on this day (Fig. 4a–c). Intriguingly, in larvae treated with > *80–100 *ng/mg, hyperactive behavior was detected 48 h after application (Fig. 4d). Moreover, there were clear differences in mobility patterns 48 h after feeding between controls and animals treated with > *40–60 *ng/mg and > *80–100 *ng/mg. Control animals showed relatively constant activity patterns over the whole observation period whereas treated larvae revealed an alternation between high and low activity phases.

### Locomotion analysis

**Figure 5:**
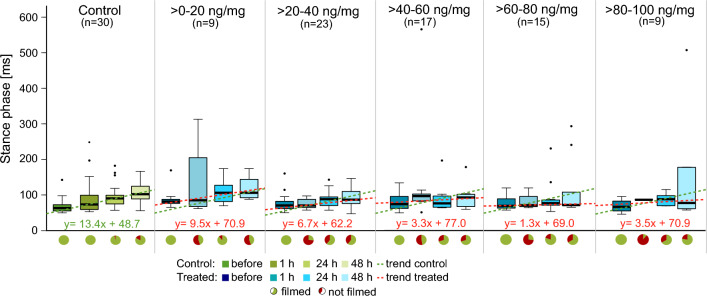
Analysis of stance phase duration in *C. carnea* larvae at different time points before and after treatment with flupyradifurone or control solution. Each graph shows the development of stance phase duration over time, for the control group and five treatment groups. The control group is depicted in different shades of green, while the treatment groups are shown in shades of blue. The dashed green lines represent the trend line of the medians in the control group, depicted in all plots, while the red dashed lines are the trend lines of the median values in the pesticide-treated groups. The pie charts below represent the fractions of animals that could be evaluated (green, walking in a straight line for at least three strides) and the ones that were not evaluated (red, either did not walk straight or no three steps without interruption). Sample size varies between the different treatment groups (see “Methods” for further information). In control animals, we found a clear and linearly increasing trend for stance phase duration. The pesticide-treated animals, by comparison, showed an increase in the stance phase duration that was up to 10-times smaller.

**Figure 6:**
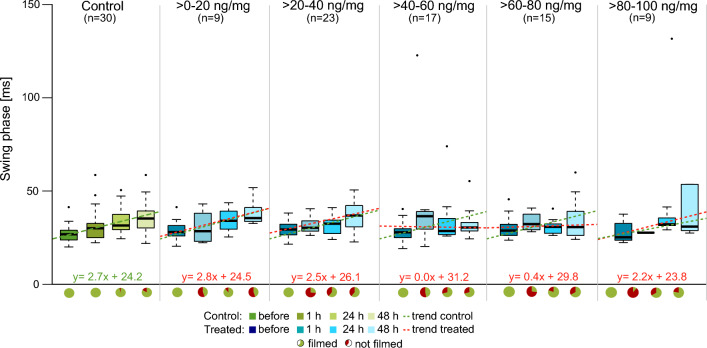
Analysis of swing phase duration in *C. carnea* larvae at different time points before and after treatment with flupyradifurone or control solution. Each graph shows the development of swing phase duration over time for the controls and the five treatment groups. The control is depicted in shades of green, while the treatment groups are shown in shades of blue. The dashed green lines represent the trend line of the median values in the control group, depicted in all plots, while the red dashed lines are the trend lines of the medians in the pesticide-treated groups. The pie charts below represent the fractions of animals that could be evaluated (green, walking in a straight line for at least three strides) and the ones that were not evaluated (red, either did not walk straight or no three steps without interruption). Sample size varies between the different treatment groups (see “Methods” for further information). Swing phase duration increased with time in all groups in a similar manner, except in treatment groups > *40–60 *ng/mg (constant swing phase) and > *60–80* ng/mg (approximately six-fold lower increase).

**Figure 7:**
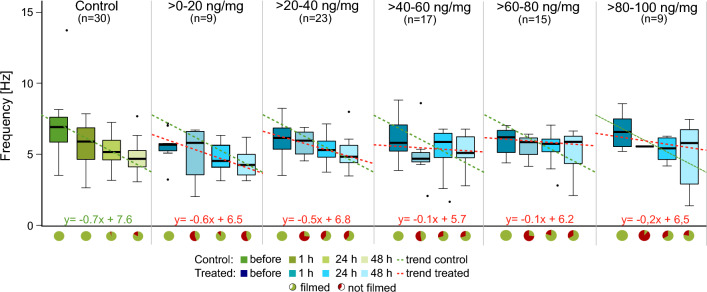
Analysis of stride frequency in *C. carnea* larvae at different time points before and after treatment with flupyradifurone or control solution. Each graph shows the development of stride frequency over time in the control group and in the five treatment groups. The control is depicted in different shades of green, while the treatment groups are shown in shades of blue. The dashed green lines represent the trend line of the medians in the control group, depicted in all plots, while the red dashed lines are the trend lines of the medians in the pesticide-treated groups. The pie charts below represent the fraction of animals that could be evaluated (green, walking in a straight line for at least three strides) and the ones that were not evaluated (red, either did not walk straight or no three steps without interruption). Sample size varies between the different treatment groups (see “Methods” for further information). Control larvae showed almost linearly decreasing stride frequencies over time, whereas pesticide-treated animals showed smaller decreases or nearly constant stride frequencies.

We further investigated the temporal changes in limb movement after intoxication. We examined stance phase, swing phase and stride frequency (Figs. 5, 6 and 7). Here, we used the same treatment groups as for the observation of the physical conditions.

Median stance phase duration of controls was shortest before feeding with 60 ms (Fig. 5). This value increased to 100 ms at 48 h after application of sugar solution, exhibiting a linear trend (y = 13.4x + 48.7). This was expected according to larval development^36,37^.

In insecticide-treated groups, stance phase duration tended to stay constant over time. Especially in the groups that had received > *40–100 *ng/mg flupyradifurone, the stance phase did not increase in length and the groups had relatively constant median values (inclines of 1.3× to 3.5×). Prior to feeding, the > *60–80 *ng/mg and the > *80–100 *ng/mg groups had approximate median stance phase durations of 70 ms like the controls, but contrary to the controls the values stayed relatively constant between 70 and 90 ms over the 48 h observation period. In the > *0–20 *ng/mg as well as the > *20–40 *ng/mg groups, smaller increases than in the controls were detected (inclines of 9.5× and 6.7× ).

The observation of swing phase duration showed similar trends as the stance phase (Figs. 5, 6). In the controls, values increased over time from 30 to 40 ms, on average. No statistically significant differences were observed between the controls and groups with applied concentrations of > *0–20 *ng/m*g* and > *20–40 *ng/mg. Only a slightly lower increase was detected in the highest concentration group. However, there were clear differences between the controls and the animals treated with > *40–60 *ng/mg and > *60–80 *ng/mg as these groups showed an almost constant swing phase duration (incline 0.0× and 0.4×, respectively) throughout the observation period. Swing phase durations were 30 ms before pesticide feeding and 30 ms after 48 h in both groups.

The final locomotion parameter examined was stride frequency (Fig. 7). Controls showed stride frequencies decreasing from 7.0 to 4.7 Hz over time (y = − 0.7x + 7.6). In groups treated with > *0–20 *ng/mg and > *20–40 *ng/mg as well as in the highest concentration group, there were decreases with smaller declines compared to the controls (inclines of − 0.2 to − 0.6×). The remaining two treatment groups (> *40–60 *ng/mg and > *60–80 *ng/mg) showed an almost constant frequency (inclines of − 0.1×). Median values observed in the first group were 5.9 Hz before and 5.1 Hz 48 h after pesticide application whereas the latter group showed a median value of 6.2 Hz in the beginning and 5.9 Hz during the last observation.

Another important fact to mention is the number of filmed animals. All controls were alive and able to walk during the first two observation periods, and only 17% of the animals were dead after 48 h (Figs. 5, 6 and 7). By contrast, in all pesticide-treated groups, a large number of animals, up to 89% in the > *80–100 *ng/mg group, were either unable to walk or died immediately after treatment. With increasing time, some of the pesticide-treated animals recovered, if still alive.

## Discussion

We demonstrated lethal and sublethal effects of flupyradifurone on *C. carnea* larvae after oral administration. Concentration-dependent behavioral changes such as trembling and coma were identified. Tracking analyses revealed acute hypoactivity, which changed to time-delayed hyperactivity in flupyradifurone-treated animals. Analysis of leg movements during locomotion showed that flupyradifurone altered the trends of stance phase, swing phase and stride frequency during larval development.

### Flupyradifurone significantly decreases long-term survival

We observed an LD_50_ of *120–200 *ng/mg (mean amount: 219 ng/larva) after 168 h (Fig. 1b). For better comparison with other studies, we here used the mean amount of pesticide fed per animal. LD_50_-values for bees vary from 1200 to 2995 ng/bee (summer in-hive) and up to 6823 ng/bee (early spring in-hive) depending on several external parameters like age, worker type or season^21,27^. LD_50_ for bumblebees is estimated within the same range at 2823 ng^38^, which is also higher than the LD_50_ we determined for lacewings. Larvae of *C. carnea* showed a 5- to 31-fold lower LD_50_ than bees and bumblebees, potentially resulting in an underestimation of the toxicity risk of flupyradifurone for lacewings, especially considering that the adult stage proved to be even more susceptible than the larvae^31^. Overall, species differences in mortality indicate a substantial problem because most risk assessments were conducted with bees and thus can massively underestimate the harmfulness to other insects^39^.

Intriguingly, we found a lower mortality rate in the > *100–120 *ng/mg (87% survival) treatment than in all other groups, including controls (82% survival, Fig. 1b). We interpret the lower mortality as the result of a concentration-dependent effect termed hormesis^40^. This dose-dependent mode of action has been described several times for flupyradifurone, for example in honeybees, resulting in higher survival rates at certain doses^26,27^. Hormesis also occurred after treatment with other nAChR-agonists in pest and beneficial insects like bumblebees^41–43^, however, the phenomenon is not fully understood.

### Flupyradifurone induces trembling and coma even at the lowest concentration applied

We showed that all flupyradifurone concentrations induced changes in physical conditions, such as coma or trembling (> *0–100 *ng/mg or > 0–184 ng/larva, Fig. 2). Similar behaviors were observed in winter bees treated with 1200 ng/bee flupyradifurone. These bees showed significantly higher rates of immobility^44^, which is consistent with the comatose behavior we observed. Tosi et al.^26^ reported abnormal behaviors that occurred at concentrations even as low as 11.1 ng/bee if the animals were fed chronically for a period of 10 days. Behavioral changes, like uncoordinated movements, immobility and trembling, have also been described after treatment with neonicotinoids like imidacloprid or thiamethoxam^29,45–48^. These results prove that the effects of neonicotinoids and butenolides are remarkably similar, even if different species are used for analyses. Nevertheless, in different species, behavioral changes are induced by different pesticide concentrations. The effects of flupyradifurone on lacewings occurred at much lower concentrations than reported for bees. As a result, lacewing larvae are more likely to exhibit behavioral abnormalities in agricultural areas treated with (presumably) bee-friendly concentrations. Therefore, they have a higher risk of being harmed under field conditions.

### Flupyradifurone delays pupation and adult emergence

Larval development was especially impaired by the flupyradifurone concentration of > *80–100 *ng/mg (mean amount 167 ng/larva, Fig. 2f). Even though no statistical differences were detectable, flupyradifurone tended to delay the developmental time course. The first pupated larvae occurred not before 216 h in this treated group, whereas the first pupation was detected after 72 h in controls, aligning with previously noticed retardation of larval development following flupyradifurone exposure in *C. carnea*^31^. Delayed pupation was reported after oral administration of thiamethoxam in *Apis mellifera* larvae^49^ whereas in the stingless bee *Scaptotrigona* aff. *depilis,* Rosa et al.^50^ reported a shortened larval stage and a prolonged developmental time of the pupal stage. Further, Wu et al.^51^ demonstrated that sublethal concentrations of neonicotinoids can affect the development of honeybees after they had been reared in pesticide-contaminated brood combs. This finding is highly interesting because it suggests that the threat to insects such as *C. carnea* during development may be even greater than to honeybees. Lacewings lay their eggs on the underside of leaves, and thus, it appears likely that the eggs come into direct contact with pesticides in cases of foliar application, exacerbating pesticide impact.

### Flupyradifurone causes acute hypoactivity and time-delayed hyperactivity

Our tracking analyses demonstrated that flupyradifurone induces changes in mobility for at least 48 h. Immediately after feeding, intoxicated animals showed hypoactive behavior (Figs. 3, 4). These results are consistent with previous studies indicating that various nAChR-agonists such as flupyradifurone, imidacloprid or thiamethoxam can lead to a hypoactive state after intoxication^15,44,46,47,52^. Beyond previous findings, our study has shown that the effects of flupyradifurone can extend over a period of up to 48 h. This provides us with further evidence that pesticides binding to insect nAChRs lead to comparable behavioral changes, regardless of their exact chemical structure. This similar mode of action in neonicotinoids and butenolides is not surprising since both substance classes share a common target receptor. We detected larvae walking in small circles (Supplementary Video V2) during the first observation period after feeding. Circular movements were also observed in other studies using flupyradifurone, imidacloprid and thiamathoxam^16,44,53^. Hesselbach and Scheiner^44^ reported that bees walked in circles, mostly between 15 and 60 min after flupyradifurone feeding. Interestingly, they found differences between long-lived winter bees and summer bees, with consequences being more severe in winter bees. They hypothesized that this effect was due to different activity of detoxification enzymes. These findings again highlight the pronounced variability of flupyradifurone effects, even within a single species. It is therefore even harder to transfer the results of one species to another without conducting additional experiments.

Over time, hypoactive behavior (1 h after treatment) was superseded by a hyperactive state in the > *40–60 *ng/mg and the > *80–100 *ng/mg groups (48 h after pesticide consumption, 100 ng/larva and 120 ng/larva, respectively, Figs. 3, 4). Such changes in mobility patterns were also observed after treatment with the neonicotinoids thiamethoxam or imidacloprid. Tooming et al.^15^ reported a gradual transition from hyperactive to hypoactive behavior over time after thiamethoxam treatment in *Platynus assimilis*. Regarding the application of increasing imidacloprid concentrations, Teeters et al.^52^ reported a transition from hyperactive to hypoactive behavior. Unfortunately, observation periods differed between all those studies, preventing a proper comparison of time-dependent changes. Nevertheless, as all studies showed the emergence of hypo- and hyperactive behaviors, nAChRs-agonists arguably induce notable changes in the mobility patterns of different insect species. These impacts on mobility, especially hypoactivity, could become a serious problem for animals in their natural habitat, as hypoactive or comatose animals are more likely to get preyed upon. Indeed, Kunkel et al.^53^ described that beetles are more often preyed upon under the influence of imidacloprid. Further, it remains unclear whether larvae are able to obtain sufficient food or find hiding places when hypoactive. Main et al.^54^ demonstrated that predators had an 84% decrease in predation success after contact with neonicotinoids. In addition, animals in a hyperactive state are likely to be impaired since they have increased energy expenditures and are probably unable to properly coordinate their actions, such as prey capture. Under these circumstances, it can be expected that larvae would perish under field conditions. In laboratory studies , the animals are provided with water and food ad libitum, temperature and humidity are controlled and there are no predators. Field conditions likely exacerbate laboratory effects, as animals are exposed to pesticides mixtures or in a chronic manner.

### Flupyradifurone affects larval locomotion

In control animals, we observed an increase of stance and swing phase durations over 2 days, according to normal larval development (Figs. 5, 6). Such increases were not detectable or minimal in pesticide-treated groups. The trend for stride frequencies was reversed, with a decrease in the controls and approximately constant stride frequencies in all treated larvae (Fig. 7). Our mobility analysis revealed different mobility patterns over time, which are in good agreement with these locomotion data. Larvae of the control were highly active at the beginning of the observation period and became less active with increasing time, whereas treated animals showed the inverse trend. These behavioral differences would appear to be caused by the altered locomotion since fast walking speeds are achieved by shortening of stance and swing phase and increasing stride frequency (and vice versa)^55^. Moreover, few pesticide-treated animals were able to walk at all. Immediately after pesticide feeding, more than half of the larvae in all treatment groups were not able to walk three step cycles in a straight line resulting in a high amount of animals standing still. Similar results were reported after honeybees were exposed to neonicotinoids^13,44^. In keeping with the above lines of argument, these effects are extremely detrimental to the animals and further suggest that they would be unable to survive under field conditions.

### Field relevance

*C. carnea*, found on all continents except Australia, serves as a natural predator of soft-bodied insects and is employed as a biological control agent in various agricultural settings, including vegetable, fruit, and nut cultivation, as well as in greenhouses^56^. This exposes *C. carnea* to potential contact with pesticides like flupyradifurone in both natural and agricultural environments through multiple exposure routes. One pathway of intoxication involves the predation of different contaminated pest animals, e.g. aphids. Estimating a daily intake of 4–34 aphids per lacewing^37,57^, flupyradifurone doses associated with various thresholds in our study (59 ng for sublethal effects; 219 ng for LD_50_) suggest that 1.7–14.8 ng and 6.4–54.8 ng flupyradifurone content per aphid could lead to sublethal and lethal effects within one day. While direct flupyradifurone data are limited, LD_50_-values for aphids exposed to pesticides with similar mode of action fall within the previously mentioned concentration range: 14.52 ng/aphid for sulfoxaflor^58^; 68.42 ng/aphid for thiamethoxam^58^; 1.84 ng/aphid for imidacloprid^59^. An additional exposure route is ingesting flupyradifurone-treated plant material. Despite their preference for small arthropods, Chrysoid larvae supplement their diet with non-prey foods like extra-floral nectar, pollen, or honeydew for nutrition^60–62^. In worst-case scenarios of various flupyradifurone residues (^21^, Table 28), quantities of treated plant material adequate to induce sublethal (> 59 ng/individual) and lethal effects (LD_50_ = 219 ng/individual) in *C. carnea* are as follows: 0.86 mg (sublethal) and 3.22 mg (lethal) for blueberry pollen (found residues: 68 mg/kg); 2.68 mg (sublethal) and 9.95 mg (lethal) for cotton nectar (found residues: 22 mg/kg); 2.81 µl (sublethal) and 10.43 µl (lethal) for oil-seed rape guttation liquid (found residues: 21 mg/L). Although these plant material amounts seem rather high at first sight, we have to keep in mind that under field-realistic conditions, lower pesticide levels may adversely impact insects due to chronic exposure and potential interactions with other pesticides mixtures^27^. Laboratory studies on bees have shown that chronic exposure to concentrations 10-times lower than those effective in one-time treatments can trigger abnormal behaviors^26,44^. All developmental stages, including eggs, are prone to pesticide intoxication, potentially due to contaminated surfaces. Adult lacewings, feeding on nectar, are also likely affected by contaminated food, a concern that is amplified by the heightened vulnerability of adult lacewings compared to larvae^31^. Consequently, the poison risk extends throughout their lifespan.

## Conclusion

Our data show that LD_50_ of flupyradifurone 168 h after oral administration to *C. carnea* larvae was impressively lower than LD_50_ in bees. We also demonstrate that the lowest used concentration of flupyradifurone induce behavioral changes like trembling or comatose animals, whereas higher levels lead to delayed pupation. It is not yet clear whether or not these effects may have further impact on adult longevity or fecundity. With our mobility analyses we identified acute flupyradifurone-induced hypoactivity that changed into time-delayed hyperactivity at certain concentrations. We further investigated changes in larval locomotion after pesticide treatment, leading to constant stance and swing phase durations and stride frequencies, as opposed to lengthened stance and swing movements and decreasing stride frequencies in controls as pupation preliminaries.

Significant lethal and sublethal consequences for lacewing larva were already observed at flupyradifurone doses of > *0–200 *ng/mg or mean amounts of 59–219 ng/larva. One may assume that these very low concentrations represent field-realistic scenarios, despite poor data availability regarding flupyradifurone residues in the field. This is especially worrying because insects have to face additional stressors in their environment enhancing the negative impact of pesticides. There is a growing body of evidence that the ubiquitous and prophylactic use of pesticides poses a serious risk to non-target organisms and ecosystem functions. This study demonstrates that it is essential to consider a broader spectrum of insect fauna representatives during risk evaluations. Considering species-specific sensitivity differences, it is essential to focus not just on honeybees to estimate pesticide impact on faunal communities. Rather it is necessary to use behavioral parameters that are comparable in a species-independent manner for future risk assessment studies. Our data further emphasize—in conjunction with many other studies—that sublethal pesticide exposure negatively influences insect vitality. Sublethal effects should not be overlooked, they may have consequences not only on life history but also on population level. With this knowledge in mind, we propose development of bioassays that include parameters describing lethal and sublethal effects in a broad range of insects to adequately assess risks concerning both agricultural production and environmental services—that are ultimately inextricable. Such an approach would help to obtain a comprehensive impression of the harmful effects of a given substance on a wide range of insects, hopefully further allowing low-level assessment of effects on the given ecosystem.

## Methods

### Animal care, pesticide solutions and feeding

We used four different concentrations of flupyradifurone (50, 100, 200, 300 ng/0.1 µl) dissolved in 50% sucrose solution, and a 50% sucrose solution as a control. These concentrations were used due to results of preliminary experiments, which showed that 50 ng of flupyradifurone was insufficient to reach LD_50_ after 48 h. We normalized the final concentrations achieved in the animals with regard to body mass, resulting in seven pesticide test groups: *> 0–20, > 20–40, > 40–60, > 60–80, > 80–100, > 100–120* and *> 120–200 *ng/mg. Nevertheless, to allow proper comparison with former literature, these concentration ranges were also converted to a mean ng/individual-value. Note that for one experimental pesticide range different animal numbers were used in various experiments (survival, behavior, mobility analysis) and therefore different corresponding ng/individual-values were obtained. Despite these differences, we chose to maintain varying ng/individual-values for each pesticide range in each experiment. This facilitates accurate assignment within our investigations and enables straightforward comparison to other studies. The stock solution for pesticide feeding was prepared as follows: 0.03 g flupyradifurone was diluted in 10 ml of 50% sucrose solution. Subsequently, all concentrations were prepared from this stock solution. For all experiments, we used flupyradifurone (Lot#BCCB1463, > 99%purity) by Sigma Aldrich (Steinheim, Germany). Flupyradifurone is highly water soluble (3200 mg/l, 20 °C, pH 7,^21^) and therefore no additional solvent (water alone suffices) was used in our experiments.

*C. carnea* larvae were ordered from “Sautter und Stepper” (https://www.nuetzlinge.de/). Each animal was housed individually in a plastic box (diameter 3.5 cm) containing a piece of paper towel as hiding place and as a rough surface for pupation. Individuals were allowed to recover from shipping for at least two days before the start of experiments. Throughout the study period, larvae were maintained at 60–80% humidity, temperatures of 24 ± 2 °C and fed aphids every two to three days (*Acyrthosiphon pisum,* Ben´s Jungle, https://bens-jungle.com/). Animals that were in poor condition (impaired walking, supine position) after the initial laboratory acclimatization days were excluded from experiments.

Before feeding pesticide and control solutions, all larvae were weighed (Sartorius, MC210P) to normalize final pesticide concentrations with respect to body mass, as mentioned above. We tried to use only animals in the second larval stage and to select individuals with approximately similar body masses. On average, the animals weighed 2.42 mg (s.e.m. ± 0.1 mg, compare Supplementary Table S1). Then, each animal was fed individually with 0.1 µl of feeding solution, that is, the different pesticide concentrations or control sugar solution. Individuals were placed on a hydrophobe, non-slippery surface, a droplet of feeding solution was applied to the surface with a pipette (Eppendorf, ResearchPlus 0.1–2.5 µl), and the larvae were carefully guided there with a fine brush. When the animal quit feeding and left, it was checked that the entire droplet had been ingested. If solution remained or the larvae took longer than 5 min to absorb the droplet, the respective animals were excluded from experiments. This procedure ensured that the amount of pesticide applied was accurately known for each individual. Previous studies had identified three sources of pesticide exposure: Contact with contaminated surfaces, contact with spray droplets, and eating contaminated food^63^. We decided to feed a specific dose of flupyradifurone directly in order to study the effects of different pesticide concentrations on the larvae as accurately as possible.

Note that, although we started all experiments with the same number of individuals, there is a variation in the final number of individuals within each experimental group. This was due to three different factors. (1) We only considered animals where we could assure a complete flupyradifurone exposure for our experiments (rate of animals per group which ingested the full volume varied between 57 and 72%). (2) After exclusion of animals that did not entirely ingest their pesticide doses (see 1), remaining animals were reorganized into distinct flupyradifurone ranges normalized to body weight, which further increased variation of animal count per experimental group. (3) Finally, over the course of several days during data acquisition, dead larvae led to a reduction in the test animals available for experiments within the specified time frame. Altogether, these parameters contributed to variations in the number of individuals in the experimental groups.

### Survival rates and analysis of physical condition and development state

First, we determined the effects of the different flupyradifurone concentrations on larval survival. Larvae were fed flupyradifurone with the above concentrations, and lethality was recorded immediately after feeding and 24 h, 48 h, 72 h, 144 h, 168 h, 192 h, 216 h, (i.e. 1, 2, 3, 6, 7, 8, 9 days) after feeding (Fig. 1b). For further experiments, only concentrations from > *0–100 *ng/mg were used (Figs. 2, 3, 4, 5, 6 and 7). Groups treated with > *100–120 *ng/mg and > *120–200 *ng/mg were disregarded for further evaluation since only few larvae were able to consume the provided pesticide solution (see above). Animals showed spasms and trembling and were unable to complete food intake. We thus decided not to apply these concentrations any further. Simultaneously to the lethality recordings, observations of physical conditions were performed. Each animal alive was observed for 20 s and assigned to one of the following categories: *vital, trembling, coma, dead, pupated, adult*. Animals in vital condition were able to walk properly and moving about; trembling animals showed uncoordinated shaking of legs, abdomen or head (see Supplementary Video V1); animals in comatose condition showed no sign of movement. To distinguish dead and comatose animals, individuals were touched with a brush, and if they showed no response after direct contact with the brush—even minimal movements of legs, antennae or abdomen—they were considered dead. Larvae were classified as pupated as soon as they began to spin their cocoon.

### Tracking analysis

Video-based analyses were performed with a sampling rate of 60 Hz using an EthoVision XT15 video tracking system (Noldus, Wageningen, Netherlands). Larvae were filmed at three different time points after feeding (1 h, 24 h and 48 h) for 4 h each. We used a Basler acA1300-60gm camera and filmed twelve individuals from above. Each animal was placed under a plastic cover (diameter 6.3 cm) that was placed on a sheet of paper. This setup was positioned on a Plexiglas board and illuminated from below by infrared spotlights (RayTEC Var2-i2-1, wavelength 850 nm). This illumination provided good contrast between background and animals (Fig. 3a) and allowed tracking of the animal silhouettes with EthoVision (compare Supplementary Video V3). Experiments were carried out in complete darkness except for the infrared illumination. Between different experiments, all insects were placed back in their housing boxes and kept as noted above, “Animal care, pesticide solutions and feeding”. After video recording, data were examined using EthoVision XT 15 tracking software to determine activity state, traveling distances and paths. For activity analysis, we defined three states that describe larval body movements independent of spatial motion of an animal’s center of gravity. For example, a grooming or trembling animal may be in a highly active or active state even if remaining in place. Assignment to highly active, active and inactive states depended on the percentage of pixels changing between the current and the previous video frame. We defined the states by thresholds: pixel changes of > 70% = highly active; < 10% = inactive and 10–70% = active. Further analyses and evaluations of raw data were then performed in Excel 2016 (Microsoft Corporation, Redmond, WA, USA) or RStudio Version 4.0.5 (The R foundation for statistical computing, Boston, MA, USA).

### Locomotion analysis

We performed video analyses using a highspeed camera (Mikrotron, MotionBlitz EoSens Mini1-1, Nikon Lens 100 mm 1:2.8 DG MACRO, Japan) to further scrutinize the effects of flupyradifurone on mobility. This allowed analysis of animals´ leg movements during locomotion. Videos were recorded at 400 or 500 Hz sampling rate, different frame rates being due to walking speed differences and recording window dependencies. To obtain videos in top view, the camera was placed above the recording area. For each video, one larva was filmed while walking at least three step cycles per leg in a straight line with a constant speed (compare Supplementary Video V4). To ensure that the animals could walk without slipping and to obtain high contrast, we used white paper as walking substrate. Each animal was filmed at least twice per day at a temperature between 24 ± 2 °C. Videos were recorded 24 h before feeding, and 1 h, 24 h and 48 h after feeding.

Data were analyzed frame-by-frame using ImageJ 1.51 s (National Institutes of Health, USA, http://imagej.nih.gov/ij). To compare locomotion of control animals with that of pesticide-treated animals, a set of standard walking parameters was used, namely, swing and stance phase durations and stride frequency^64^. These parameters have been shown to be useful to describe different walking situations like forward and backward walking^65^. With this knowledge, we decided to use these parameters for pesticide related locomotion analysis. Tarsal lift-off and touch-down were determined to calculate swing and stance phase durations. Swing phase was defined as the time between tarsal lift-off and touch down, and the stance phase was the time period during which the tarsus touched the ground and was not moving relative to the ground^66^. Mean walking speed was calculated from distance traveled during the three evaluated step cycles and the time taken for travel. Likewise, stride frequency was calculated by dividing mean walking speed by mean stride length.

### Statistics

Excel 2016 (Microsoft Corporation, Redmond, WA, USA) and R Studio Version 4.0.5 (The R foundation for statistical computing, Boston, MA, USA) were used for statistical assessments. All data were initially tested for normal distribution with a Kolmogorov–Smirnov-Test. To compare survival rates we used Kaplan–Meier survival curves and Log-Rank-Test. The different categories of physical conditions as well as the activity states were analyzed with the Chi-Square-Test. We tested the controls against the different pesticide-treated groups for each day separately. For the traveled distances per day as determined with the EthoVision XT15 Tracking System and the activity patterns we used the Kruskal–Wallis-Test for non-normally distributed data. Pairwise comparisons were performed with a Wilcoxon-Test with Bonferroni-adjusted p-value. The same procedure was carried out for the locomotion parameters stride frequency, swing and stand phase durations. Boxplots show the first and third quartiles representing 25 and 75% of the data, respectively, medians, whiskers indicating the data point no further than 1.5 times the interquartile range from the box, and outliers.

### Supplementary Information

Supplementary Video 1.

Supplementary Video 2.

Supplementary Video 3.

Supplementary Video 4.

Supplementary Tables.

Supplementary Legends.

## Data Availability

The datasets generated and analyzed during the current study are available from the corresponding authors on reasonable request.
