# Supplementary material for: Influence of the pesticide flupyradifurone on mobility and physical condition of larval green lacewings
Source: Sci Rep. 2023 Nov 13;13:19804. doi: 10.1038/s41598-023-46135-7 (PMC10643709; doi:10.1038/s41598-023-46135-7)
Supplement: Supplementary file 6 — Supplementary Legends. [file 41598_2023_46135_MOESM6_ESM.docx]

**Video V1:** Video of a trembling larva after pesticide intoxication. Shown is the behavior at original speed and in slow motion (10x).

**Video V2:** Example of circular movements of lacewing larvae after intoxication (speed 8x).

**Video V3:** Exemplary tracking with EthoVision (speed 8x).

**Video V4:** Example of a walking larva filmed with a high-speed camera. First the animal is shown with original speed, then in slow motion (20x).
